# Hospital and Institutional News

**Published:** 1917-01-27

**Authors:** 


### HOSPITAL AND INSTITUTIONAL NEWS.

### THE KING AND THE LONDON HOSPITAL SATURDAY FUND.

THE accounts of the Hospital Saturday Fund (London) for 1916 were closed on January 8. In spite of the hindrances and limitations imposed by the strain of war circumstances, the total income is nearly £10,000 greater than that of 1915, and about £16,000 more than that of 1914; for the year 1916 the General Fund amounted to over £46,000, and the three subsidiary committees (Surgical Appliances, Distribution, Ambulance) accounted, in addition, for more than £10,000 between them. The chairman of the Fund, Sir T. Vezey Strong, has submitted these figures to the King, and has received from Lord Stamfordham the following reply:—" Your letter of the 10th inst. has been laid before the King, who was interested in the particulars it gave of the result of the 1916 collections made on behalf of the Hospital Saturday Fund. In thanking you for this information, I am further commanded to express His Majesty's gratification at the successful results achieved during the past year, thanks to the untiring efforts of the honorary workers of the Fund." The sentiments so admirably and concisely conveyed in this letter express the feeling of the Metropolis so thoroughly that it is unnecessary to do more than quote them. May the Fund progress in the future as splendidly and surely as it has done in the last three years!

### KING MANOEL AND MILITARY ORTHOPÆDICS.

KING MANOEL, who is evincing the keenest interest in the problem of disabled soldiers and the establishment and development of curative workshops at various great centres, will pay another visit to Cardiff during February to inspect the progress made at the Prince of Wales' Hospital for Limbless Sailors and Soldiers. The Welsh scheme is being enthusiastically taken up, and the work is concentrated at the Mansion House. King Manoel has appointed Mr. Lynn Thomas, C.B., to act for him in regard to the Cardiff Orthopædic Hospital, in the same way that other eminent surgeons are representing him at Leeds and Liverpool. Lieut.-Colonel Robert Jones, F.R.C.S., has accepted the position of consulting surgeon to the Welsh hospital, and has been promoted to the temporary rank of colonel in the Army Medical Service. The question of the relation of the Army medical authorities to the efforts put forth at the newly established orthopædic hospitals is engaging the Government's attention, and the probability is that general control will be given to Sir Alfred Keogh, with a director working under him.

### SHORTAGE OF RESIDENTS IN HOSPITALS.

TWENTY hospitals in London and the provinces are advertising for resident medical officers. Practically all of these institutions invite applications from medical women; indeed, one or two of the vacant posts are open to women only. The salaries offered range from £60 to £250, and as a general rule the smaller institutions offer the larger salaries. These facts indicate that the difficulty in obtaining residents is an increasing one, and that the women practitioners who three years ago had to seek hospital appointments are now being sought with great persistence. An appointment at the Bristol General Hospital, where apparently the possibility of obtaining a male resident is considered not quite out of the question, may be held for " three months under the scheme of the War Office whereby recently qualified medical men may hold three months' posts as residents after joining the R.A.M.C."; and at St. Bartholomew's Hospital, Rochester, there is a vacancy, with a salary of £110, for a clinical assistant, ineligible for the Army, who may be either qualified or unqualified. At a small special hospital in London a house physician is offered £250 a year, while at several other institutions the salary is evidently, and very sensibly, considered a matter for negotiation, and no mention of it is made in the advertisement.

### A HOSPITAL NOT A "NATIONAL INDUSTRY."

THE connection between the hospitals and the State in Australia has curious manifestations. We recently recorded the forthcoming arrangement under which some hospitals must, willy-nilly, obtain their supplies of drugs and dressings from a State depôt; we now have to refer to a curious state of affairs in which the various State Governments found themselves unable to help the hospitals to obtain an adequate supply of fuel. It appears that during a recent strike only " national industries " could be supplied with coal and firewood, and as by some remarkable reasoning public hospitals could not be included in this description they found themselves in a serious plight, many of them being reduced to using rather precarious supplies of odd wood to keep the boiler fires alight. It appears that the Navy Department was responsible for supplies, and, apparently under pressure, an undertaking was given to help six of the largest public medical charities in each capital city in Australia, the institutions being chosen by the Department. What happened to the smaller institutions is not recorded.

### THE HOME AT RICHMOND HILL.

STUDENTS of architectural and administrative problems will have noted with interest that Mr. Gilbert Scott's plans for the new " Star and Garter " Home for Paralysed Soldiers at Richmond have been criticised on the grounds that they are not in keeping with the magnificent surroundings, and that they resemble a barracks—the most hideous of modern buildings as a rule. While expressing, at the moment, no opinions on the justness of these criticisms, we may point out that confusion of mind which has been the bane of much recent hospital architecture. Modern hospitals of any size were being built some twenty years ago without much regard for economy; and the architect was expected to inform the public by means of façades, pillars, and porticos of the huge sums which the public had subscribed. The recent reaction against this extravagance has had two useful results. It has led to a keener study of the relation between administration and design, called the experienced manager into the architect's office; and it has also led to a return to simplicity. We may quote the new surgical block of the Bristol Royal Infirmary as an agreeable example of both. But as design may run to seed in ornament, it may degenerate into something that is repulsive. The plans at Richmond are apparently criticised on the latter ground. Mr. Gilbert Scott is, no doubt, quite capable of defending his own designs, but we may venture to remind him and his critics on the Richmond Town Council that beauty in architecture, and especially in hospital architecture, is mainly the result of proportion, and proportion is not only the relation of structure to function, but also of structure to ornament. Over-simplicity is as much to be avoided as ornamentation for its own sake.

### A MINERS' MISTAKE IN SOUTH WALES.

The workmen engaged at the Llewellyn, Merthyr, and neighbouring collieries have agreed to contribute weekly one penny per man to the Merthyr General Hospital on condition that the institution is open to all the local panel doctors, that two representatives of the medical staff sit on the executive, that a governor be appointed for every £5 contributed, and that three executive members for each contributing body be appointed. These terms would be described as stiff were they relevant, and it is not surprising that decision has been deferred. But they are not even relevant. The men apparently wish to drive a bargain. It is the duty of the Merthyr General Hospital to remind them that a voluntary hospital can never adopt that attitude with regard to any subscriptions whatever. Once such an attitude were admitted, endless problems would arise which would place all contributors, including the miners, in a very uncomfortable position. The amount of work done on their behalf would have to be computed, and experience justifies the prospect that the treatment of miners "costs" more to the hospital than their contributions warrant. The hospital, in this way, would be doing bad "business." But to do business, good or bad, is not a voluntary hospital's function. Its function is to help the sick in time of trouble.

### CAN A HOSPITAL ACCEPT CONDITIONAL CONTRIBUTIONS?

That a hospital may be able to carry out its proper functions, people, of their charity, give voluntary subscriptions. To subscribers and donors some privileges may be given; but they are not sold. There is, and can be, no bargain about it. The miners, like the rest of the community, presumably wish to do their share in enabling the Merthyr General Hospital to continue its work. Let them give so far as generosity prompts them—but "representation" and a hand in the staff arrangements, and so on—these are not to be sold. We hope the hospital committee will make this clear, even if it declines the proffered "gift" in order to do so.

### A HANDSOME LIBRARY FOR PATIENTS.

The Base Hospital at Leicester has received a library of 1,000 selected books from the West End Association for the Entertainment of Wounded Soldiers, and two of its members have given the bookcase. In congratulating the Association on the selection made, the Mayor of Leicester remarked that books were essential to supplement newspapers, for man could not live by newspapers alone. This last statement is more commonly admitted in theory than acted on in fact; but it is always worth making, since technical newspapers nowadays are almost the only class of journal which can never escape from the standard set by serious readers. As to the selection, we have not seen the list; but we may recall the action of a public man of letters who, when he presented books at all to any hospital or institution other than a library, invariably insisted that the men and women in the institution should make their own list, in addition to which he would add a percentage of his own. "They know from what books they get most," he used to say; "I may bias their choice, but must not make it." So handsome a gift as this at Leicester is worth much as an example merely.

### A SYMBOL OF GROWTH.

The rapid development of the East Suffolk and Ipswich Hospital is symbolised in the fact that the old entrance, with its gates, has had to be dismantled, owing to the great amount of traffic, including ambulances, which has come, particularly since the war, to the hospital's doors. "Gates worthy of the institution" will be asked for at the end of the war, when it will be possible to dignify, as well as to use, the wider approach created by the sacrifice of the original entrance. Voluntary hospitals depend so much on tradition that little relating to their past should be sacrificed, and therefore, if the old gates have any use at all, they should be employed somewhere else about the hospital, for their present use as the principal entrance gives them a sentiment which, perhaps, neither their design nor material alone could do.

### THE NEED FOR MEDICAL ASSESSORS IN COMPENSATION LITIGATION.

The urgent need for all legal tribunals to avail themselves of the power to call upon the services of an approved medical adviser to the Court in compensation and other cases requiring a survey of conflicting medical testimony is well illustrated afresh by a case heard on January 18 at the Merthyr County Court. A certain watchman in the employment of Guest, Keen and Nettlefolds threw the shackling of a blocking chain from between two trains in December 1915, and immediately thereafter suffered an hæmoptysis. Six months later the man died, and the widow brought an action for compensation on the ground that his death was due to strain caused by the act of throwing the shackle.

One doctor, called for the plaintiff, is reported as saying that the throwing of a chain might have caused the bursting of a blood-vessel, resulting in the patient's death. Another attributed death to aneurism, due to strain. Two others, however, who had the advantage of seeing the organs *post mortem*, deposed that there was a tuberculous cavity in the left lung, and that death was due to tuberculosis. Notwithstanding this evidence, the plaintiff got a verdict for £300, with costs. If the report in the *Western Mail* is accurate, it cannot but be suspected that a medical assessor would have advised the Court in a sense likely to be fatal to the contention that death was due to aneurism or strain; on the facts as disclosed in that report of the evidence, the presumption in favour of tuberculosis is overwhelming.

### AN AD MISERICORDIAM APPEAL SUCCESSFUL.

The extremely necessitous position of the Royal Albert Hospital, Devonport, was a matter which came under consideration at a recent meeting of the Council of the Plymouth Hospital Sunday Fund, which met for the purpose of allocating the total amount collected by the Fund among the various institutions. The committee recommended an award of £115 to the Royal Albert Hospital, but the claims of that institution to a larger share were strongly urged, on the grounds that it is now practically £1,200 in debt. The committee decided, therefore, to increase the grant to £130. The other apportionments were as follows: South Devon and East Cornwall Hospital, £303; Homœopathic Hospital, £65; Eye Infirmary, £20; Ear and Throat Hospital, £20; King Edward VII. Dispensary, £2 2s.; and Dental Hospital, £2.

### THE NEW GOVERNMENT AND INFANT WELFARE.

A great forward movement in child welfare is being launched with the earnest advocacy of Lord Rhondda, the new President of the Local Government Board. The problem of conserving the infant life of the nation so impressed its vital importance upon Lord Rhondda, even in the first week of his responsibility at Whitehall, we are told, that he has decided to bring in a Bill as soon as Parliament assembles to extend and increase the powers and obligations of the local authorities in regard to maternal and child welfare. An inquiry which has been made by the Local Government Board into the quality and quantity of this type of work undertaken by local governing bodies throughout the country has laid bare great inequalities, due too often to lack of enlightened enterprise, or to a desire for so-called economy. Lord Rhondda's desire is to legislate so that the example of the best shall become the standard of all the authorities, and to effect this desirable change it may be necessary to invest the authorities with further powers and, perhaps, to incorporate in the measure an element of compulsion. Points upon which Lord Rhondda is known to have fixed as vital are the enlargement of the army of trained health visitors and midwives, the provision in every locality of maternity centres, in addition to a liberal provision of lying-in institutions, with a sufficiency of surgical wards, and provision, likewise, for the proper sustenance of mother and child during the all-important periods. In order that legislation on these lines may be got through Parliament with all possible speed, the President of the Local Government Board is understood to be willing to agree that his department shall meet any increase of expenditure necessary in the national interest.

### MINERS' SUBSCRIPTIONS DOUBLED.

On the whole it is impossible to read without satisfaction the speech of the Lord Provost at the recent annual meeting of the Edinburgh Royal Infirmary. After indicating the work by stating that the daily average number of patients in hospital had been 908, he was able to add that the ordinary and extraordinary income together had not only met the ordinary and extraordinary expenditure, but that a little over £5,000 remained in hand. How far an unusual run of legacies accounted for this is, of course, a question, but we note with special pleasure the agreement of the workers in the Lothian and Fifeshire oil-works and coal and shale mines to double their regular contributions. These had, therefore, reached a sum of £6,000. Such a position has been accompanied by negotiations for an increase in the representation of the working class upon the board of management. The committee advised the court to add five workmen's representatives. The Lord Provost explained that statutory powers were necessary to carry this change into effect, but that it was intended that the required provision should be included in the first Municipal Bill which is being promoted in Parliament.

### DEVELOPMENTS AT LETTERKENNY.

After our comments on the extraordinary happenings at Letterkenny, and the subsequent letter from the Irish Medical War Committee published in the last issue of The Hospital (January 20, 1917, page 330, the latest proceedings of the Board of Guardians there are both interesting and amusing. It will be remembered that the Nationalist majority on the Board of Guardians terminated the appointment of a temporary medical officer, who was over military age, in favour of a very young Irish doctor, eligible for the R.A.M.C. The Irish Local Government Board, whose instructions had been openly flouted in the matter, holds that neither the dismissal of the middle-aged Unionist nor the installation of the youthful Nationalist is legal, and there has been talk of law proceedings to determine several points connected with the dispute. At the last meeting of the Guardians it happened that the Nationalists were poorly represented, and the normal Unionist minority found itself in temporary control. They accordingly refused to pay the young doctor's salary, and also certain other fees; and they ordered immediate payment of the older doctor's claim, which had previously been refused by the Nationalists. If the spectacle of open disloyalty in Ireland were not so tragic in relation to the world war for freedom and right, these developments would turn the whole dispute into screaming farce.

### DEATH OF MISS MARY WARDELL.

The death of Miss Mary Wardell, at the ripe age of eighty-five, removes one who without ostentation or self-advertisement devoted her time and her means to a particular form of institutional charity. It was in 1884 that Miss Wardell, who had already worked for many years among the poor of London, founded the Mary Wardell Convalescent Home for scarlet-fever cases at Stanmore, in Middlesex. Since then nearly 6,000 patients have passed through her institution, which gained the patronage of Queen Alexandra and of the Duchess of Albany. Although primarily a charitable home, Miss Wardell soon saw the need for accommodation during the convalescent stages of scarlet fever in patients who were able to pay reasonable fees. She accordingly devoted part of her house to the reception of such patients, making thereby profits which doubtless helped to keep the charitable home (which was in the same grounds) on its legs. In both branches of the work she filled a gap in the hospital treatment of the sick which no one else—in or near London—cared to fill; and for this, as well as for her devoted personal service for so many years, her memory will long be held in affectionate memory by her patients.

### DUMFRIES PARISH COUNCIL AND THE MOORHEADS HOSPITAL.

A somewhat acrimonious controversy, conducted with what at a distance seems to be an unnecessary warmth, is taking place between the Dumfries Parish Council and the management of the Moorheads Hospital. The latter institution is a charity for the respectable poor, in which the inmates remain in many cases for years at a time. About the end of last year an aged inmate developed, or is alleged to have developed, habits of indecency; whereupon the managers of the hospital turned him out and called upon the Parish Council to maintain him in the poor-house. The man himself came to Moorheads from some other parish, where he has now lost his domicile by the length of his residence at Moorheads. The Parish Council, with some reason, do not see why the ratepayers of Dumfries should be saddled with indigent paupers imported from a distance by the Moorheads Hospital, and maintain that the facilities for looking after him at the latter institution are, or should be, as great as those at the poor-house. Into the squabble as to the merits of the accommodation provided by the two institutions, and into the difference of opinion as to the exact mental and physical condition of this particular patient, it profits little to enter. But, on broad principles, the contention that the burden to the rates of Dumfries created by the action of the Moorheads Hospital is a distinct hardship has our sympathy.

### IRREGULARITIES AT THE WEST RIDING ASYLUM

At an inquest held recently at the West Riding Asylum, Wakefield, on a young female inmate who died of scalds, certain evidence was given which calls for action on the part of the management. It appeared that, contrary to the rules, it has long been a practice there to fill a bath with hot water and to allow the women who clean the day-room boards to fill their buckets from the bath. The young woman who formed the subject of the inquest apparently went to the bath for this purpose and fell into it, sustaining scalds which proved fatal. The evidence of the nurses was " given in such an unsatisfactory way and was so contradictory " that the jury were unable to say which evidence they believed. At all events, the junior nurse upon whom the immediate responsibility in connection with the incident fell asserted that she had been permitting this practice of filling water-buckets from the bath for six months; and that neither of the two senior nurses had ever told her that she was doing anything wrong. This allegation seems to have been denied, or partly denied, by the charge nurses. At all events, a breach of the rules was clearly of frequent occurrence, and it may be hoped the incident will render such ill-discipline conspicuous by its absence in the future.

### THE ST. JOHN AMBULANCE BRIGADE HOSPITAL.

One of the best arranged and best maintained hospitals, built on the hut system, in the Pas de Calais district is the St. John Ambulance Brigade Hospital at Etaples. It is, as anyone who has visited it will testify, a model of good order, and the reputation of the work carried out by the medical and nursing staff is excellent. An appeal is being issued by the Order of St. John for further funds to carry on this particular branch of its organisation, which has been in full work for the last sixteen months. When originally founded it was not contemplated that its services would be required for so long a period, and the funds contributed were thought to be sufficient to carry the work to a conclusion. But like many of the predictions in connection with the war, this estimate has had to be revised, and although, of course, faith will be kept with those who endowed wards and the donors for the provision and upkeep of beds in the hospital named after them, the time has come when the exchequer of the hospital must be strengthened. There is very little doubt that the original donors, having as it were a stake in the undertaking, will come forward again in many instances, as will probably the several county and colonial organisations that endowed wards. Mr. Evelyn Cecil, M.P., the Secretary-General of the Order, who signs the appeal, issued from St. John's Gate, Clerkenwell, should have a good response to his letter.

### CLEARING HOUSE FOR ORGANISERS.

Out of the social upheaval of the last two years has emerged a body of organisers of all kinds of projects of a charitable or business nature, chiefly in connection with conditions caused by the war or movements for its vigorous prosecution. A committee has been formed, containing, amongst others, the Earl of Chichester and Earl Percy, with Captain Percy Creed as chief organiser, to create a Clearing House and Intelligence Department for the organiser; its first task, as is learned from a circular recently issued, is to collate the work and requirements of "associations, leagues, and similar societies." But the new society appears to direct its solicitude more to the interests of the organiser than the organised, and expresses its intention to help "square pegs" into "square holes," to guide those who are wishful to organise in the right direction, and to help those who want employment in the "higher grades of organisation work." The 2,000 or more institutions, the essential features of the work of which are to be found in "Burdett's Hospitals and Charities," may form a trifling field of activity for those who are collating information on behalf of the new Clearing House; but we rather think that most of these societies will be already provided with its own organiser, and will find some difficulty in appreciating the helpfulness of any new society which offers to lure him away to a "higher grade of organisation work."

### PRECAUTIONS IN THEATRE WORK.

THOSE of our readers who were interested in the December Question in our *Insitutional Worker Supplement*—and we have received so many replies to it that we are certain it aroused widespread interest—will be pleased to hear that the subject of precautions to prevent swabs, instruments, etc., from being left inside patients at operations has recently engaged the attention of the American Association of Obstetricians and Gynæcologists Dr. Weiss, of Pittsburg, read a paper at the last session of this society in which he dealt with this very topic; and many of the best known operators in the United States took part in the discussion. Pretty well every speaker had a different system. Dr. Weiss was very emphatic about the personal responsibility of the surgeon, and the wrongness of any attempt on his part to delegate it to anyone else. Dr. Keefe deprecated swabs altogether, advising rubber sheets in their place. Incidentally two of the speakers told strange stories concerning the presence of relatives during an operation, a matter upon which Dr. Weiss had touched in his paper. One of these speakers mentioned that on one occasion the husband of the patient insisted on being present during the operation. Afterwards he confided to the surgeon that he watched very carefully "to see if my wife quit breathing or looked as though she was going to die; and if there were any indications of it, I had my revolver ready to shoot you." Operative surgery must be an exhilarating pursuit in America, if incidents such as this are at all common!

### THE SMALLPOX PERIL.

WHAT the medical officer of health calls "an unpleasant incident" has happened at Preston, where there have been two cases of smallpox. The two cases had no connection, and the cause of infection could not be traced. The medical officer says: "From their situation it was necessary that both cases should at once be removed, and the want of any local hospital provision made itself acutely felt. The Ducker Hospital at Holme Slack is now in even a more dilapidated condition than when visited by your sub-committee, and affords no adequate accommodation for either patients or nurses. Fortunately, through the courtesy of the Secretary to the County Joint Hospitals Board, I was enabled to obtain their early removal to the Elswick Smallpox Hospital, where Dr. Fisher, medical officer to that institution, at once undertook their treatment. As a nurse was urgently required at Elswick, and could not be otherwise obtained, I asked for volunteers from our own Isolation Hospital, and obtained the services of Nurse Prescott, who was at once conveyed to Elswick, where she is at present in charge. There has been one other suspicious case, which after careful observation proved not to be smallpox. The sudden appearance of smallpox in the town—which may any day be repeated—only shows the danger which must exist to an unvaccinated, or imperfectly vaccinated community, and the necessity of having immediately at hand some means of isolation. Pending the erection of a hospital for the town I would advise that arrangements should—if possible—be made with the authorities of the Elswick Hospital whereby cases may be received from Preston, as they occur, without the necessity for further conference. Although the past outbreak has been cut down, another may at any time occur—especially bearing in mind the constant influx of military recruits into the town—and this may not be so satisfactorily dealt with. In safeguarding the town from the danger of an outbreak I had the benefit of the advice and experience of Dr. McEwen, of the Local Government Board, who visited, and remained some days in the town."

### HEALTH VISITORS' SALARIES.

THE Local Government Board is frowning upon low salaries for health visitors. In the case of the Preston Corporation the Board intimated, with reference to a proposed appointment of an additional health visitor, that the initial salary offered was lower than that usually offered, and the Corporation has now decided that the commencing salary shall be £78, rising by annual increments of £6 10s. to £97 10s., with an allowance of five guineas for uniform.

### A STRICT DISCIPLINARIAN.

NEWS has lately reached this country of the recent resignation of Miss Payne, for many years Lady Superintendent of the Wellington Hospital, New Zealand. A sound tribute is paid to her in the local newspapers, one of which remarks: "Her strict regard for discipline and routine may not have been appreciated by the younger nurses, but when they rose to positions of authority themselves . . . they were always proud to say that they were Miss Payne's nurses." "The discipline which finds a young man or woman raw and leaves him sulky is one thing; the discipline that leaves him proud of his instructor is another." Lately, having been asked to advise on a school for a difficult boy, we sought his own opinion of the place which he was leaving. "No one made us work," he said, "except Mr. Blank, and he was the only one we had any regard for." As the utterer of this remark was alleged to be rebellious and lazy, there can be no fear that he was insincere in what he said. So long as the junior members of a class or staff realise that the rule which is imposed achieves some object beyond its own oppressiveness they will not in the long run resent its incidence. The example of Miss Payne shows that loyal co-operation is the fruit of a strict but intelligible discipline.

### INFANT WELFARE IN CORNWALL.

The difficulties in the way of framing anything like an ideal maternity and infant welfare scheme for a county like Cornwall—with its few concentrated areas and many sparsely populated rural districts—are obvious. These obstacles, however, have not prevented the County Medical Officer (Dr. Clarke) from formulating a plan which has in it the elements of success. This scheme, which was unfolded last week and adopted by the governing committee, is estimated to cost about £900 a year; it provides for the immediate appointment of three whole-time health visitors at salaries and expenses amounting to £450, and an additional three later on. These visitors will work in towns where the district nurses have sufficient to do without health visiting. In the rural areas—at any rate for the present—it is proposed to rely largely upon the services of the district nurses, whose areas, if too unwieldy, will be made more workable under an arrangement with the County Nursing Association, so that the nurses may act as health visitors as well. A capitation grant of 2s. 6d. in respect of each birth is the suggested payment by the County Council to the Nursing Association, which will certainly not be able to make any profit out of the scheme. More midwives will have to be provided, and, if the county is to be really covered, twenty-three new districts will have to be opened up. Without the co-operation of the Nursing Associations the Council would find their task too expensive, for in these scattered rural districts a nurse-midwife would starve if she had only her fees to live upon. There are sixteen Nursing Associations in the county not affiliated to the County Association, and eight of these have expressed themselves as willing to enter into arrangements with the Council for the joint services of their nurses. The seven remaining Associations replied that they could take no part in the work.

### THE SUPPLY OF NURSES IN WALES.

With a comparatively small income of between £400 and £500 a year, the South Wales Nursing Association is doing work of real national importance. Forty-seven nurses have been trained under its auspices, and forty-three are now at work in the villages of the constituent counties; many, as Major Ewen Maclean observed at the Association's annual meeting at Cardiff, have given up much to discharge their duties in lonely rural places of the Principality. More might be done if more money were forthcoming, for the demand for the services of the nurses still exceeds the supply. With the help of the Marchioness of Bute's endowment fund (which it is hoped to bring up to £5,000), together with scholarship grants from the County Council and an enhanced grant in aid from the Local Government Board—which already contributes £130—the training of additional candidates will be undertaken. There is urgent need of more nurses, for some of the counties in West Wales stand very badly in regard to puerperal mortality and first-year deaths among infants. So far as Cardiff itself is concerned big extensions may be looked for following Colonel Bruce Vaughan's recent visit to Bradford in order to see the inner workings of the model maternity and child-welfare scheme in operation there.

### A PHARMACISTS' FIELD AMBULANCE.

A new corps, whose membership is open to pharmacists, dispensers, laboratory and other assistants, is being formed at Birmingham under the command of Lieutenant-Colonel J. T. J. Morrison. Known as the Birmingham Chemists' Field Ambulance (Volunteer) Corps, the corps has for its aim the training and drilling of its members with a view to their attachment to the Warwickshire Command as a complete unit of the Royal Army Medical Corps. A course of lectures on physiology, anatomy, and first-aid is being arranged, and the new movement is already understood to have the support of Mr. Wilfrid Hill, Mr. H. Buckingham, Mr. Radford, and Mr. Corfield, in addition to that of the medical officer of health, Lieutenant-Colonel Robertson, and the Commandant of the Warwickshire Volunteers.

### THIS WEEK'S DRUG MARKET.

The downward tendency in the prices of some of the most commonly prescribed synthetic products continues. Thus salicylic acid, salicylate of soda, acetylsalicylic acid, salol, and phenacetin are all cheaper, and the general feeling is that the downward movement will continue. In the case of phenacetin the fall in price will probably be slow, but there is no doubt that the output is increasing considerably; the other products named have now reached prices which, in view of the restricted supplies of raw materials, will hardly allow of a further rapid decline, and any reductions that take place will probably be small. A drug which appears to have disappeared from the market is storax; this is one of the ingredients of compound tincture of benzoin, and it looks as if the authorities may be compelled in due course to allow of some alteration in the formula for this tincture; possibly balsam of Peru could be substituted for storax without great disadvantage—but, of course, this could not be done without the sanction of the General Medical Council. Cocaine continues very scarce. All classes of henbane are becoming scarcer. The prices of petroleum jellies, both white and yellow, are advancing in consequence of the high freights from America. Quinine is in rather better demand, and the price is well maintained. Honey is again dearer.

